# The relationship between work-life balance and psychological well-being: an empirical study of metro rail travelers working in the information technology sector

**DOI:** 10.3389/fpsyg.2024.1472885

**Published:** 2025-01-20

**Authors:** K. D. V. Prasad, Mruthyanjaya Rao, Rajesh Vaidya, Kottala Sriyogi, Shivoham Singh, Ved Srinivas

**Affiliations:** ^1^Symbiosis Institute of Business Management, Hyderabad, India; ^2^Symbiosis International (Deemed University), Pune, India; ^3^Human Resources, CMR University, Bangalore, Karnataka, India; ^4^Symbiosis Institute of Business Management, Nagpur, India; ^5^Thiagarajar School of Management, Madurai, India

**Keywords:** occupational stress, self-acceptance, work-life balance, autonomy, environmental mastery

## Abstract

**Objectives:**

To investigate the relationship between work–life balance and the psychological well-being of metro rail travelers working in the information technology sector. The study also examined occupational stress as a pathway between work-life balance and psychological well-being. The study also investigated the impact of occupational stress and work–life balance on the psychological well-being of metro travelers who work in the information technology sector, modeling lower- and higher-order constructs.

**Methods:**

A quantitative survey method was used, and the data were gathered from information technology employees who frequently travel on Metro Rail to commute to the office and return home when the COVID-19 pandemic peaked in India in 2022. A structured questionnaire was developed, and a link was provided to the IT sector employees visiting almost all the metro stations in Hyderabad, an Indian Metro, to measure 8 reflective constructs. The data were gathered via random sampling, and the questionnaires were randomly distributed to the different IT sector companies. The valid responses of 500 participants were analyzed for structural equation modeling. The eight reflective constructs in the study are occupational stress, the 3 constructs of work–life balance—“work interference with personal life, personal life interference with work and work–personal life enhancement”—and the four constructs of psychological well-being autonomy, self-acceptance, positive relations, and environmental mastery.

**Results:**

The SEM results for the lower-order constructs indicate that the impact of occupational stress on psychological well-being was statistically significant (*p* < 0.005), as were the two constructs of psychological well-being, environmental mastery, and self-acceptance (*p* < 0.001; *p* < 0.05). With respect to the impact of the work–life balance constructs, the impacts of the WIPL, WPLE, and PLIW work–life balance constructs were statistically significant (*p* < 0.05; *p* < 0.001, respectively) for all four psychological well-being constructs. Occupational stress partially mediated the relationship between work–life balance and psychological well-being, as both the direct and indirect effects were statistically significant when the higher-order constructs work–life balance and psychological well-being were tested. The direct effects of occupational stress and work–life balance on psychological well-being are statistically significant (*p* < 0.05, *p* < 0.001).

**Conclusion:**

The authors suggest framing policies to mitigate occupational stress and enhance the psychological well-being and work–life balance of employees in the information technology sector.

## 1 Introduction

### 1.1 Stress

The stress from employment perceived by employees is occupational stress or job stress. An Austrian-born endocrinologist used the term “stress” to describe an unpleasant expression (Selye, 1956). The main reasons why an employee experiences stress or work overload, role ambiguity, a lack of peer support, a lack of social support, and role conflict can be stress. Stress is present in every individual; however, only the degree of stress differs among individuals. Uninterested work, disliked supervisors, and work and role overload can cause stress in employees (Prasad et al., 2015). However, limited stress is needed, which can work as a catalyst and enhance employee performance. The authors reported several side effects of occupational stress, such as gastrointestinal disorders (Huerta-Franco et al., 2013), musculoskeletal disorders and effects on mental health (Li et al., 2021), hypertension, type 2 diabetes mellitus and lipid disorders (Djindjic et al., 2012), psychosocial disorders (Buunk et al., 2013; Vaca-Auz et al., 2024) and socioemotional behavioral issues.

### 1.2 Work–life balance

How well an employee is allocating and managing his/her time between work and family, managing relations, personal growth, and managing his/her hobbies is work–life balance. Some employees juggle their time to meet the demands of the employer and personal life well. Sometimes working class battles to accomplish work–life balance if the office settings are problematic and working more than the stipulated time. Aruldoss et al. (2021) studied the impact of work–life balance and the mediating effects of occupational stress by surveying 445 respondents in a cosmopolitan city in southern India. The authors reported partial mediation of occupational stress and the impact of work–life balance on the quality of work–life of employees. Kelly et al. (2020) reported that over one-third of respondents experienced job stress and burnout, with academic struggles and work–life balance issues being major issues. Workload was the leading factor contributing to these issues. Decreased quality of life, work–home conflict, and emotional imbalance are some of the consequences of employees not having work–life balance (Hosseini et al., 2024). The productivity of an employee can be influenced by quality of work life and work–life balance (Bhende et al., 2020). Work and family demands and resources, personality antecedents, emotional behavior, and loss of interest in work are associated with not having work–life balance (Brough et al., 2020). Effect on job autonomy, diminished performance, job satisfaction and organizational citizenship behavior are some of the consequences of work–life imbalance (Haar and Brougham, 2022). Paje et al. (2020) investigated the impact of compressed workweek (CWW) on job stress, work-life balance, and productivity in the Philippines. With 350 respondents from Metro Manila, it was found that CWW implementation reduces stress, enhances work–life balance, and improves productivity. The findings could benefit employers considering institutionalizing CWW.

### 1.3 Psychological well-being

An employee’s positive condition and state of mind with happiness is psychological well-being. If an employee feels that he/she is happy and satisfied with available resources, this indicates that the person’s psychological well-being (PWB) is high. PWB is the extent to which a person experiences positive feelings and emotions of happiness (Diener, 2000). “Carl Ryff, a psychologist, has developed a 6-factor model of psychological/Eudemonic well-being (Ryff and Keyes, 1995; Ryff et al., 2010). The state of mind and perceived stress can impact people, resulting in anxiety and mood disorders (Gladstone et al., 2004), which are related to the psychological well-being of an individual. Effects on cognitive functioning, health, social relationships and emotional imbalance are some of the issues associated with psychological well-being (Huppert, 2009). Researchers have examined the link between self-compassion and psychological well-being in Turkish adults and the effects of psychological resilience, fear of COVID-19, and psychological discomfort. It was also discovered that psychological distress mediates the relationships among COVID-19 anxiety, fortitude, and mental health (Hatun and Kurtça, 2023). Self-compassion was shown to have substantial indirect benefits on psychological well-being through mediating variables. The results indicate that self-compassion decreases psychological distress and boosts resilience, which in turn increases well-being (García-Campayo et al., 2024). Foster et al. (2020) explored workplace stressors, psychological well-being, resilience, and caring behaviors and examined relationships and differences in resilience on the basis of sociodemographic characteristics. The study revealed a moderately strong relationship between workplace resilience and psychological well-being, with consumer/career-related stressors being the most stressful challenge for nurses.

Carol Ryff, a renowned psychologist, developed the first systematic model of psychological well-being, which remains one of the most scientifically verified and empirically rigorous models today. During that time, current psychological theories of well-being lacked empirical rigor, as they had not been and could not be tested. Ryff combined philosophical and scientific perspectives on well-being, identifying recurrence and convergence across various theories and forming her new model of well-being. Carol Ryff’s psychological well-being model emphasizes multidimensional aspects rooted in Aristotle’s Nichomachean Ethics, focusing on living virtuously rather than just happiness or positive emotions and promoting a balanced, whole life. The six factors of Carol Ryff’s psychological well-being are self-acceptance, personal growth, purpose of life, positive relationships with others, environmental mastery and autonomy. Carol Ryff’s psychological well-being model offers a valuable framework for life analysis and improvement, but media coverage is lacking, suggesting that Ryff may need better marketing staff.

Work–life balance and psychological well-being are higher-order constructs. The psychological well-being construct has six subdimensions (Ryff and Keyes, 1995; Ryff et al., 2010), whereas work–life balance has three subdimensions (Hayman, 2005). However, not a single study has modeled these constructs as higher-order constructs and reported the results. The authors reviewed the literature on occupational stress and psychological well-being, highlighting the need for more studies to model work–life balance and psychology as higher-order constructs. This study explored the relationships between work-life balance, psychological well-being, occupational stress, and work-life balance and the mediating role of occupational stress in this relationship. Therefore, the authors carried out this study to investigate the impact of occupational stress, work–life balance (modeled as a higher-order construct with three subdimensions), and psychological well-being (modeled as a higher-order construct with 4 subdimensions). This empirical study also tested the hypotheses with both lower- and higher-order constructs. This study is unique in its nature.

Higher-order constructs, also known as hierarchical component models in structural equation modeling (SEM) (Lohmöller, 1989), provide a framework for researchers to model a construct on a more abstract dimension (referred to as a higher-order component) and its more concrete subdimensions (referred to as lower-order components). This paper provides a method for evaluating higher-order constructs in SEM via repeated indicators and two-stage approaches, addressing common confusion in researchers’ reliability and validity assessments. The study uses the SEM example to illustrate the specification, estimation, and validation of reflective-reflective higher-order constructs, providing guidance for scholars, marketing researchers, and practitioners. The testing of the constructs with both the higher- and lower-order constructs and their assessment will provide a much clearer impact on the constructs and will have more practical implications for including organizational practices to mitigate stress and enhance employee work–life balance and psychological well-being in general and the IT sector in particular.

### 1.4 Workplace spirituality, stress, and PWB

Garg (2017) explored the relationships between workplace spirituality and employee commitment, job satisfaction, and work-life balance satisfaction, addressing the conceptual and empirical gaps in the field of human resource management. This study explores workplace spirituality via necessary condition analysis, revealing Karma Capital as a key factor in employee commitment, job satisfaction, and work-life balance satisfaction, with further investigation through a correlation matrix and regression analysis. Saxena et al. (2020) investigated the correlation between workplace spirituality and work stress levels among offshore and onshore Indian oil and gas industry employees. The study involved 202 respondents, mainly managers and supervisors from oil and gas companies such as ONGC, Cairn India, RIL, BPCL, and IOCL. The respondents were chosen because they perceived similar work stressors. Statistical tools such as the mean, t test, correlation, and multiregression were used for data analysis. The study revealed that workplace spirituality negatively impacts stress for onshore employees, whereas community and gratitude are insignificantly associated with stress. Stressful conditions and excessive specialization may hinder employees from cherishing their community and gratitude. The study suggests that normal working conditions offer sufficient opportunities for workplace spirituality. The authors explored the relationships between workplace spirituality dimensions (swadharma, authenticity, lokasangraha, sense of community) and work-to-family enrichment, examining the mediating and moderating effects of psychological and social capital. A study of 387 Indian hospitality industry female employees was conducted, and the data were analyzed for reliability, validity, and multicollinearity. This study examined the relationship between workplace spirituality and work–life balance, controlling for demographic variables and moderating gratitude and psychological and social capital effects (Garg and Sharma, 2024). The study revealed that Indian workplace spirituality significantly influences WTF enrichment, with dimensions such as psychological and social capital playing a significant role in meditating and moderating this effect. Therefore, workplace spirituality can reduce stress and enhance psychological well-being.

### 1.5 Research gap

The authors critically reviewed the literature and could source more literature on the effect of occupational stress and psychological well-being in general, work–life balance in particular. However, past studies have not modeled work–life balance and psychology as higher-order constructs to study the overall and individual impacts of work–life balance and occupational stress constructs on psychological well-being. The authors examined the impact of a higher-order construct of work–life balance on the higher-order construct of psychological well-being and each lower-order construct of occupational stress and work–life balance effect on each lower-order construct of psychological well-being. The mediating role of occupational stress in the relationship between work-life balance and psychological well-being was also examined and reported.

### 1.6 Research questions

Is there any relationship between the occupational stress and psychological well-being of metro travelers who work in the information technology sector?

Is there any relationship between work–life balance and the psychological well-being of metro travelers who work in the information technology sector?

Is occupational stress a mediator of the relationship between work–life balance and the psychological well-being of metro travelers who work in the information technology sector?

### 1.7 Objectives

- “To study the relationship between work–life balance and the psychological well-being of metro travelers working in the information technology sector
- “To study the impact of occupational stress on the psychological well-being of metro travelers working in the information technology sector”
- “To study the mediating effects of occupational stress on the relationship between work–life balance and the psychological well-being of metro travelers working in the information technology sector”

## 2 Literature review

### 2.1 Occupational stress and work–life balance

In a cross-sectional study, Mu et al. (2024) evaluated the prevalence of burnout, work overload, and work–life imbalance according to various specialties and investigated the impact of these factors on burnout among Chinese medical personnel. The authors noted that a work–life imbalance is linked to burnout and work overload. In Bulgaria during the pandemic, general practitioners’ levels of professional burnout and their contentment with the harmony between their personal and professional lives were examined by Kilova et al. (2024). Reevaluating health policies and involving more hospital care doctors on the front lines of the fight against severe infectious diseases are necessary in light of the unrealistically high levels of burnout experienced during the COVID-19 pandemic, which can be attributed to a disrupted work–life balance. Policy makers, healthcare administrators, and other stakeholders may find the findings of this study useful for understanding the major influences on general practitioners’ stress levels during the COVID-19 pandemic. The relationships among work-related stress, sources of solace, and job satisfaction were investigated by Rawat and Dani (2024). Meal servers employed in the hotel and catering industries provided information. An investigation via hierarchical regression analysis revealed that the factors that had the greatest effects on occupational stress were job demands, job control, and the connection between work-life balance practices. The results also demonstrated that a lack of work–life balance practices, high job expectations, and little job control contributed to higher levels of stress. In their 2024 study, Maharani and Tamara (2024) examined the relationships between work-life balance, occupational stress, and turnover intentions, using job satisfaction as a mediating factor. The financial services industry’s work–life balance and occupational stress phenomena are linked to intentions to leave. The intention to leave and work–life balance are directly correlated. The relationship between work–life balance and intentions to leave is negatively mediated by job satisfaction.

### 2.2 Occupational stress and psychological well-being

The authors assessed the degree of perceived stress, anxiety, depression, and occupational stress. Using a cross-sectional study design, the author also evaluated the relationships between these mental health parameters. in Qatar’s faculty. The faculty stress index, or FSI, was measured. Higher FSI scores were significantly correlated with an increased risk of at least moderate levels of stress and self-perceived depression (*p* < 0.001). Faculty from humanities colleges were more likely to have at least moderate levels of anxiety, but faculty over 50 years of age were less likely to have at least moderate levels of depression (*p* = 0.034) (Hammoudi Halat et al., 2024). The effects of stress management intervention techniques on enhancing the well-being of IT professionals in the workplace were investigated by Iswarya et al. (2024). The results of the structural equation modeling show that the well-being of IT professionals is significantly increased by mindfulness, resilience, psychological interventions, managerial grid training, and wellness programs. On the other hand, programs for conflict resolution, work role training, and behavioral intervention have yielded non-significant results [Cheng et al.’s (2024) study examined the relationship between productivity and job satisfaction in relation to employees’ psychological well-being who work in hazardous environments]. At baseline, there was a greater cross-sectional risk of serious psychological distress among participants exposed to the home-work environment (HWE), and this risk increased as the number of HWE elements increased. Over time, exposure to HWE was linked to a greater chance of developing depression and psychological well-being. Using a cross-sectional quantitative survey, Amoadu et al. (2024) examined whether psychosocial work factors could predict the psychological health and risky driving behaviors of long-distance bus drivers in Ghana. Among these drivers, psychological well-being and safety incidents are directly and significantly predicted by job demands and job resources. Furthermore, there was a strong negative correlation between drivers’ psychological health and safety incidents. Safety incidents and psychological well-being were found to be negatively correlated with the psychosocial safety climate (PSC) and positively but not significantly correlated with each other. Despite PSC theory’s premise, PSC has a negative and significant association with job resources.

Thus, the following hypotheses are formulated:

H1: Occupational stress has a statistically significant effect on the psychological well-being of information technology employees.

H1-1: The impact of occupational stress on autonomy is statistically significant.

H1-2: The impact of occupational stress on environmental mastery is statistically significant.

H1-3: The impact of occupational stress on positive relationships is statistically significant.

H1–4: The impact of occupational stress on self-acceptance is statistically significant.

### 2.3 Work-life balance and psychological well-being

Rahim et al. (2020) examined the impact of work–life balance (WLB) on career satisfaction and psychological well-being among staff at an open distance learning (ODL) university in Malaysia, examining the moderating effects of supervisor and family support. The study revealed that work–life balance (WLB) significantly impacts career satisfaction and psychological well-being, with supervisor and family support not moderating this relationship. Yusuf et al. (2022) investigated the relationship between work-life balance and the well-being of graduate students. This study explored work–life balance and well-being issues among a diverse group of graduate students, including master’s, doctoral, full-time, part-time, on-campus, and online students from various disciplines. The authors reported that social cognitive factors influence the impact of work–life balance on the psychological well-being of students. Althammer et al. (2021) evaluated a three-week online self-training intervention teaching mindfulness as a cognitive–emotional segmentation strategy and its effect on psychological well-being in the context of work–life balance. Growth curve analyses revealed positive effects on psychological detachment, work–family conflict, and work–life balance satisfaction, with low segmentation preferences causing stronger intervention effects. Using the European Working Conditions Survey, Emre and De Spiegeleare (2021) investigated the effects of commuting time on work–life balance and psychological well-being. The study also explored the impact of long commutes on employee commitment and well-being, using ‘conservation of resources’ theory and job demands-resources approaches, highlighting negative outcomes. The authors reported a negative relationship between commuting time, commitment, and well-being, with work–life balance mediating these effects and autonomy acting as a buffer against these effects. Therefore, the following hypotheses are formulated:

H2: The impact of work–life balance on the psychological well-being of information technology employees is positive and statistically significant.

H2-1: The impact of work interference with personal life on the psychological well-being dimensions of autonomy, environmental mastery, positive relations, and self-acceptance is positive and statistically significant.

H2-2: The impact of personal life interference with work on the psychological well-being dimensions of autonomy, environmental mastery, positive relations, and self-acceptance is positive and statistically significant.

H2-3: The impact of work personal life enhancement on the psychological well-being dimensions of autonomy, environmental mastery, positive relations, and self-acceptance is positive and statistically significant.

Saraswati and Lie (2020) used data from 250 diverse employees to study the role of work–life balance and work pressure in predicting employee psychological well-being. The Ryff scale, Work-Life Balance Checklist, and Tilburg Work Pressure Questionnaire were used to measure psychological well-being (PWB). Work–life balance (WLB) and work–positive (WP) impact PWB, with WLB being the dominant factor. Abdul Jalil et al. (2023) investigated the impact of job insecurity on psychological well-being, with work-life balance as a mediator. Job insecurity has a negative effect on work–life balance and psychological well-being. Work–life balance has been linked to higher levels of psychological well-being. This promotes the notion that work–life balance is a key mediator in the link between job instability and psychological well-being. These findings suggest that job security enhancement can improve individuals’ psychological well-being through work–life balance. Yayla and Eskici İlgin (2021) determined the relationships of nurses’ psychological well-being with their coronophobia and work–life balance during the COVID-19 pandemic via a descriptive, correlational and cross-sectional study. The COVID-19 epidemic has had a significant effect on nurses’ work–life balance and psychological well-being. Their COVID-19 phobia was medium to moderate. Neglecting life was the most significant predictor of nurses’ psychological well-being, followed by coronophobia and work–life balance, accounting for 75% of the variance. Ravikumar (2023) investigated the relationship between occupational stress (OS) and psychological well-being (PWB) in HCWs and police personnel during a pandemic, examining the role of positive psychological capital and the emotional quotient. Respondents’ positive psychological capital (PPC) characteristics, including faith in their abilities, performance, and resilience, have helped them manage pandemic stress and maintain their psychological well-being.

The authors investigated the potential predictors of job satisfaction, namely, work–life balance and job stress. Working from home, work-life balance, and work stress all had substantial direct and indirect effects on job satisfaction according to the PLS-SEM results. Working from home as a new work schedule can help Indonesian employees remain satisfied with their jobs in their current workplaces (Irawanto et al., 2021). Working from home might be a good indicator in a collectivist environment and should be taken into consideration by the company. However, there will not be a “one size fits all” solution, even though remote and hybrid working will grow in popularity for non-manual employment in the postpandemic future. Offices will not entirely vanish, and traditional work patterns will persist. Work done by hand will keep things the way they are even with more demands. Employers’ attention to workers’ in the new normal, WLB will strive to enhance performance and inspire workers.

In their analysis of the factors influencing work–life balance, the authors investigated how long workdays and job burnout affect workers’ mental health. The PLS-SEM results show that employee well-being and work-life balance are significantly impacted by job burnout. Employee well-being is significantly impacted by work–life balance (Vyas, 2022). Work–life balance has the ability to moderate the relationship between employee well-being and job burnout. Kolo et al.’s (2024) study examined the relationship between employee performance in commercial banks in Abuja, Nigeria, and work-life balance and succession planning. The PLS-SEM results reveal that succession planning has a positive and statistically significant effect on employee performance in banks. However, work–life balance variation has a non-significant effect on employee performance.

Anglim and Horwood (2021) investigated the effect of COVID-19 on the psychological well-being of adults in Australia. All six dimensions of psychological well-being are examined. The authors reported the negative effects of the COVID-19 pandemic on adults’ psychological well-being and subjective well-being. Bagheri et al. (2022) examined the relationship between self-efficacy and quality of life and the mediating role of psychological well-being in elderly people in Tehran, Iran. The SEM results reveal that the hypothetical model of this study has a good fit in the study sample. Thus, self-efficacy has a direct positive relationship with both psychological well-being and quality of life. Furthermore, psychological well-being has a direct positive effect on quality of life. It was also discovered that psychological well-being is indirectly related to quality of life via self-efficacy.

### 2.4 Hypotheses for higher-order constructs

H3: Occupational stress is statistically significant and impacts the psychological well-being of IT sector employees.

H4: Work–life balance is statistically significant and impacts the psychological well-being of IT sector employees.

H5: Occupational stress mediates the relationship between work–life balance and psychological well-being.

## 3 Theoretical framework

**Figure 1:**
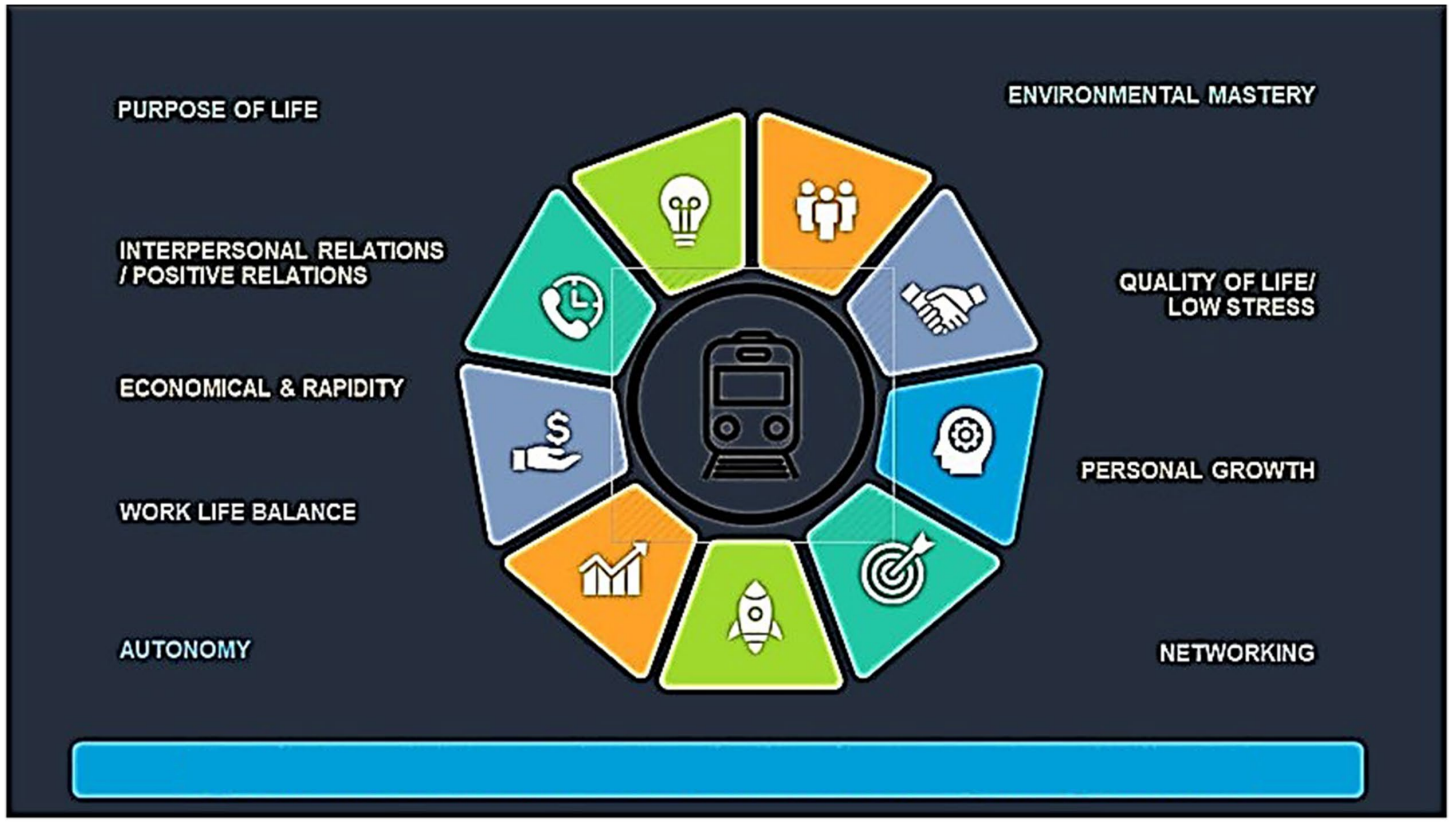
Theoretical framework.

**Figure 2:**
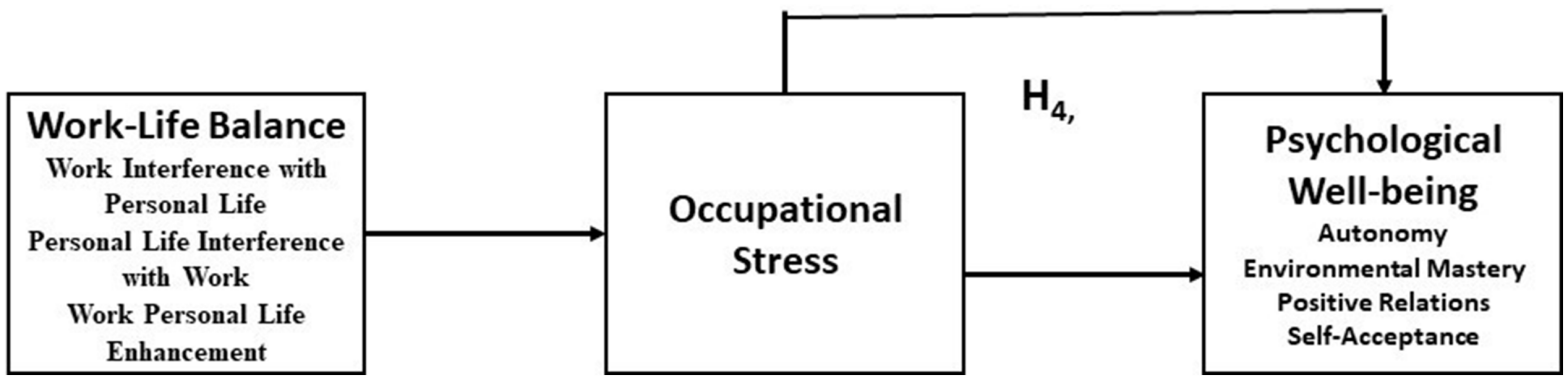
Theoretical mediation model and relations among variables (authors creation) adopted from Metselaar et al. (2022).

The theoretical framework was formulated following the models of Prasad et al. (2016), who modeled occupational stress; Hayman (2005), who developed and validated the work–life balance scale; and Ryff and Keyes (1995) and Ryff et al. (2010), who developed the structure of the shortened version of the 18-point psychological well-being scale. The authors’ theoretical framework is provided in Figure 1, and the mediation model is presented in Figure 2.

*Occupational stress*: a person’s state of mind or perceived stress from employment

Work–life balance: The ratio of the time you spend working to the time you spend with friends, family, and engaging in hobbies. It is a multidimensional concept with work interference with personal life, personal life interference with work and work-related personal life enhancement.

*Work interference with personal life (WIPL)*: Factors associated with work that influence an individual’s personal life and measure the impact of work on an employee’s personal life (Hayman, 2005).

Personal life interference with work (PLIW): This gauges how aspects of an employee’s personal life affect their work or how their personal life affects their work (Hayman, 2005).

*Work personal life enhancement (WPLE)*: How the factors related to work can enhance personal life. This may include employees’ work environment, personal policies, and flexible work, which can have a positive impact on their personal life.

*Self-acceptance*: An individual with a positive attitude.

*Environmental mastery*: A person can effectively manage his or her daily schedule while also using the extra time available for personal growth.

*Positive relations with others*: A person’s capacity for closeness, love, and reciprocation to build meaningful connections with coworkers or other members of society.

*Autonomy*: A person who is independent is not subject to political, social, or group pressure and is able to control and regulate his or her conduct.

On the basis of the review and considering the characteristics of the study, the following hypotheses were formulated.

## 4 Data analysis

### 4.1 Sampling technique and justification of sample size

A well-designed questionnaire was utilized to measure eight reflective constructs, with the first section focusing on respondents’ demographic profiles and the second section addressing questions related to these constructs. The questionnaire was prepared and published on Google Forms. The researchers visited approximately 50 metro stations in Hyderabad. The travelers were requested about their sector of employment. The Google form link was shared with metro travelers randomly with those who work in the information technology sector. A pilot study was conducted with 100 IT employees; a seven-point Likert-type scale was used, and a pretest was used to determine questionnaire suitability. Four management and IT experts and an English consultant examined the content. To avoid bias, the questionnaire was distributed to different IT companies where employees work. The questionnaire link was shared with 20,000 employees working in the IT sector industry and those who travel in Metro Rail. A total of 552 responses were received, and 52 records were not considered for the study because they were complete. The sample included 257 male employees and 243 female employees. The demographic characteristics are as follows: graduates, 312; postgraduates, 160; and others, 28; software engineers, 227; 53; technical heads, 66; project managers, 34; and others, 120.

### 4.2 Determination of sample size

Since the populations of the banking, media, and IT sectors are not known, the sample size for the unknown populations was calculated via the Cochran (1977) formula. This formula indicates that 384 is the necessary sample size. Another aspect of sample size is that having a sample size of 50 + 5X (x is the number of questions/items), which is another school of thought (Gaskin, 2019). There are 33 items in 5 reflective components in our study. As a result, 50 + 5*33 = 215 is the minimum sample size needed to apply structural equation modeling to the data; however, the 500 valid replies that are available far exceed this need. For SEM analysis, our sample size is larger than the threshold sample indicated by Wolf et al. (2013). Table 1 depicts the demography of the study variables.

**Table 1:** Study variables with their outer loadings.

Variable	Item description	Factor loadings
Occupational stress: Cronbach’s alpha 0.92, CR 0.917, AVE 0.582		
OS1	“Conditions at work are unpleasant or sometimes even unsafe“	0.78
OS2	“I feel that my job is negatively affecting my physical or emotional well-being”	0.83
OS3	“I have too much work to do an/or too many unreasonable deadlines”	0.79
OS4	“I find it difficult to express my opinions or feelings about my job conditions to my superiors.”	0.76
OS5	“I feel that job pressures interfere with my family or personal life. “	0.74
OS6	“I feel that I have inadequate control or input over my work duties. “	0.68
OS7	“I receive inadequate recognition or rewards for good performance. “	0.78
OS8	“I am unable to fully utilize my skills and talents at work.”	0.73
Work interference with personal life Cronbach’s alpha 0.911, CR 0.949, AVE 0.924		
WIPL1	“My personal life suffers because of work”	0.90
WIPL2	“My job makes personal life difficult”	0.92
WIPL3	“I neglect personal needs because of work”	0.87
WIPL4	“I put personal life on hold for work”	0.88
WIPL5	“I miss personal activities because of work”	0.87
Personal life interference with work Cronbach’s alpha 0.912, CR 0.921, AVE 0.744		
PLIW1	“My personal life drains me of energy for work”	0.88
PLIW2	“I am too tired to be effective at work”	0.82
PLIW3	“My work suffers because of my personal life and “	0.89
PLIW4	“It is hard to work because of personal matters.”	0.85
Work personal life enhancement Cronbach’s alpha 0.902, CR 0.949, AVE 0.815		
WPLE1	“My personal life gives me energy for my job”	0.92
WPLE2	“My job gives me energy to pursue personal activities”	0.88
WPLE3	“I have a better mood at work because of personal life”	0.93
WPLE4	“I have a better mood because of my job”.	0.89
Autonomy Cronbach’s alpha 0.921, CR 0.900, AVE 0.751		
AUT1	“I tend to be influenced by people with strong opinions”	0.89
AUT2	“I have confidence in my own opinions, even if they are different from the way most other people think.”	0.89
AUT3	“I judge myself by what I think is important, not by the values of what others think is important.”	0.82
Environmental mastery Cronbach’s alpha 0.897 CR 0.905, AVE 0.761		
EM1	“The demands of everyday life often get me down.”	0.89
EM2	“In general, I feel I am in charge of the situation in which I live.”	0.91
EM3	“I am good at managing the responsibilities of daily life.”	0.84
Positive relations Cronbach’s alpha 0.912, CR 0.9892, AVE 0.734		
PL1	“Maintaining close relationships has been difficult and frustrating for me.”	0.83
PL2	“People would describe me as a giving person, willing to share my time with others.”	0.87
PL3	“I have not experienced many warm and trusting relationships with others.”	0.87
Self-acceptance Cronbach’s alpha 0.911, CR 0.911, AVE 0.776		
SA1	“I like most parts of my personality.”	0.86
SA2	“When I look at the story of my life, I am pleased with how things have turned out thus far.”	0.91
SA3	“In many ways I feel disappointed about my achievements in life.”	0.84

Source: Primary data processed.

### 4.3 Sample size specification

For multivariate analyses such as factor analysis, Anderson and Gerbing (1984) suggested a sample size of 200–400 with a free parameter ratio of 5:1. This suggests that only one indication or statement requires a sample. Additionally, 50 + 5x, where x is the number of statements, was employed in accordance with the SEM analysis criterion given by James et al. (2009) and Gaskin (2019). According to these requirements, a sample size of 215 is needed for the 33 questions in the current empirical study. For this empirical investigation, the legitimate response rate of 500 subjects exceeded the necessary sample size. Moreover, the sample size is larger than that recommended for SEM analysis by Wolf et al. (2013).

### 4.4 Power analysis

With an alpha of 0.05, a power analysis was performed via SPSS version 29 to evaluate the study sample’s power (Faul et al., 2007). The sample’s standard deviation was 1.27. With an effect size of 0.82 and an actual power value of 0.915 for the sample size of 390, the results revealed a substantial and robust association between the variables. Accordingly, the study hypotheses can be tested with a sample size of *N* = 390 (Kyriazos, 2018; Goulet-Pelletier and Cousineau, 2018).

### 4.5 Measurements

The occupational stress construct is based on “The Workplace Stress Scale™ © 1978 The Marlin Company, and the American Institute of Stress” (AIS, 1978), which has 8 items (Tommasi et al., 2022). The psychological well-being scale is based on (Ryff and Keyes, 1995; Ryff et al., 2010), and this study measured four subdimensions—autonomy, environmental mastery, positive relations, and self-acceptance—and each subdimension has 3 items. The work–life balance scale is based on the model developed by Hayman (2005). This scale has 3 subdimensions: “Work Interference with Personal Life (WIPL),” 5 items; Construct 2, “Personal Life Interference with Work (PLIW),” 4 items; and Construct 3, “Work Personal Life Enhancement (WPLE),” 4 items. The study has 33 items to measure 8 reflective constructs. The demographic characteristics included 290 (58%) men and 210 (42%) women with diverse educational backgrounds. The age groups of the employees by year were 20–30,230 (46%), 31–40,160 (32%), 41–50 58 (11.6%) and > 50 years 51 (10.4%). The respondents also included software engineers (45%), technical heads (11%), testing heads (7%), team leaders (10.6%), project managers (5.4%) and others (21%).

### 4.6 Factor analysis

The factor analysis distributed the 33 variables into 8 components. A Kaiser–Meyer–Olkin (KMO) sample adequacy measure of 0.911 indicates that the data are appropriate for additional study. Eight components accounted for 80.290% of the total variance, exceeding the threshold and suggesting a value of 60% (Hair et al., 2011). To ascertain whether the variables are uncorrelated and whether the correlation matrix of the observed variable is an identity matrix, Bartlett’s test of sphericity is performed. When the correlations between the variables are considerably different from zero, as indicated by a Bartlett test value <0.001, the data are suitable for additional investigation (Chin et al., 2008).

## 5 Results

The theoretical framework and authors’ research model were tested by subjecting the data to structural equation modeling analysis via IBM AMOS version 28. The authors assessed inner and outer measurement models. The study has 8 reflective constructs with 33 indicators. Several researchers have used AMOS to assess the absolute path coefficients in several psychological and behavioral studies using normal and non-normal data (Hair et al., 2013). The lower-order model and higher-order models were tested separately.

The results of the measurement model, structural model assessment, hypothesis testing and mediation and moderation analyses are presented in the following section. The standardized regression weight loadings were > 0.7 for all the loadings except one construct (0.68); however, this item was retained for analysis because the average construct loading was >0.7. The composite reliability values are >0.7, indicating internal consistency.

### 5.1 Measurement model analysis of the lower-order constructs

The hypothesized measurement model consisted of 8 reflective constructs: occupation stress (1); psychological well-being (4); autonomy, self-acceptance, positive relationships and environmental mastery; and work–life balance (3). Work interference with one’s personal life, work-related personal life enhancement, and personal life interference with work were assessed via the maximum likelihood method, which yielded acceptable results. The model fit index is “CMIN/df = 1.911,” which is less than 2 (Tabachnick et al., 2013). CFI 0.968 > 0.95 (Hu and Bentler, 1999); NFI 0.936; IFI 0.968; TLI 0.964 > 0.95 (McDonald and Marsh, 1990; Sharma et al., 2005); SRMR = 0.030 < 0.050 (Byrne, 2013; Diamantopoulos et al., 2000); RMSEA = 0.043; PClose 0.998 > 0.05 (Hu and Bentler, 1999) are within the acceptable ranges, indicating an excellent model fit.

The average variance extracted (AVE) was examined to assess convergent validity (Table 1). The AVE values for all eight reflective constructs are >0.5, the threshold value, indicating convergent validity in the model (Hair et al., 2011). The discriminant validity (Table 1) values are >0.7 for all the constructs, indicating that there are no validity issues in the model. The discriminant values, which are indicated in bold in Table 3, were greater than the intercorrelation values between the latent constructs in each column (Fornell and Larcker, 1981). However, this method of assessing discriminant validity has been questioned by researchers; therefore, a new way of assessing discriminant validity, the heterotrait–monotrait (HTMT) ratio, was used. All the ratios fell below the necessary threshold value of 0.85 when evaluated via the HTMT (Table 2, Henseler et al., 2015), confirming discriminant validity. Convergent and discriminant validity are measured by assessing composite reliability (CR), average variance extracted (AVE), and correlations among the latent configurations. The composite reliabilities for all the variables are >0.7 and are acceptable (Nunnally and Bernstein, 1994).

**Table 2:** Discriminant validity.

	OSTR	WIPL	WPLE	PLIW	AUTN	SAC	PL	ENVM
OSTR	**0.763**							
WIPL	−0.070	**0.887**						
WPLE	−0.101*	0.447***	**0.903**					
PLIW	−0.141**	0.270***	0.265***	**0.863**				
AUTN	−0.077	0.373***	0.402***	0.236***	**0.867**			
SAC	−0.030	0.526***	0.456***	0.426***	0.326***	**0.879**		
PL	−0.091†	0.467***	0.364***	0.331***	0.272***	0.467***	**0.857**	
ENVM	−0.009	0.693***	0.566***	0.261***	0.488***	0.678***	0.380***	**0.872**
HTMT Analysis								
OSTR								
WIPL	0.069							
WPLE	0.094	0.426						
PLIW	0.129	0.255	0.242					
AUTN	0.070	0.345	0.374	0.216				
SAC	0.026	0.491	0.423	0.394	0.294			
PL	0.081	0.433	0.338	0.305	0.239	0.419		
ENVM	0.011	0.650	0.525	0.236	0.443	0.619	0.339	

Values in bold are square root of Average Variance Extracted. OSTR, Occupational stress; WIPL, Work interference with personal life; WPLE, Work personal life enhancement; PLIW, Personal life interference with work; AUTN, Autonomy; SA, Self-Acceptance; PL, Positive relations; ENVM, Environmental mastery.

### 5.2 Lower-order structural model assessment

**Figure 3:**
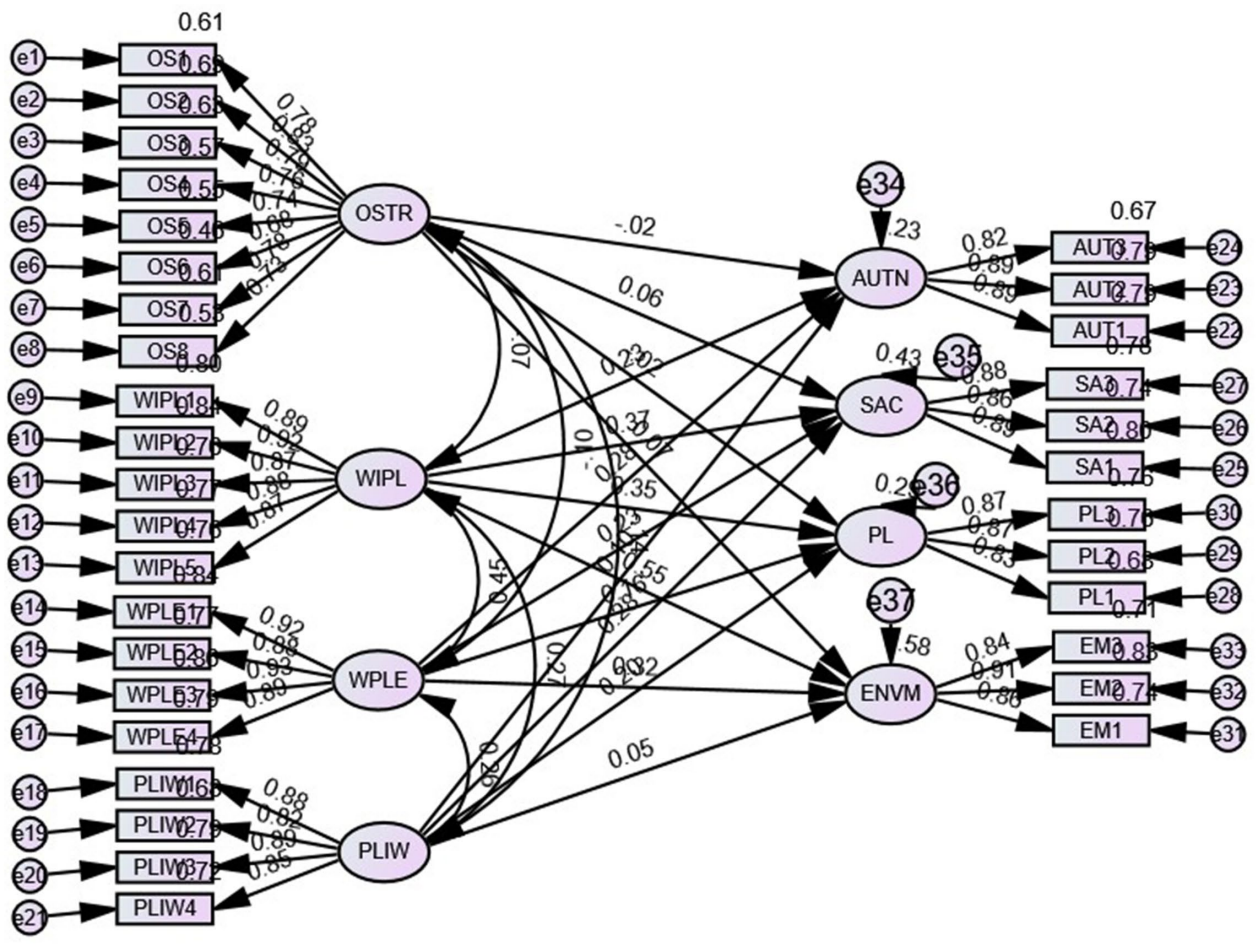
The structural model with relationships among the constructs. Source: Primary data processed. OSTR, Occupational stress; WIPL, Work interference with personal life; WPLE, Work personal life enhancement; PLIW, Personal life interference with work; AUTN, Autonomy; SA, Self-Acceptance; PL, Positive relations; ENVM, Environmental mastery.

The model fit indices CMIN/df = 1.933, CFI = 0.960, TLI = 0.955, IFI = 0.960, NFI = 0.963, SRMR = 0.042, RMSEA = 0.048 and PClose = 0.827 indicate excellent structural model fit. The squared multiple correlations (R^2^) of 0.23 for autonomy, 0.43 for self-acceptance, 0.29 for positive relations and 0.58 for environmental mastery indicate that occupational stress, work interference with personal life, work personal life enhancement, and personal life interference explain 23% of the variance in autonomy, 43% of self-acceptance, 29% of positive relations and 58% of environmental mastery (see Figure 3).

### 5.3 Model fit

When researchers use SEM, one of their primary concerns is whether their model fits well overall. The idea of fit is arguably the most misinterpreted topic in SEM, although most authors are aware that they must report model fit statistics and that reviewers depend on them to make “make or break” decisions on submissions. Few researchers obtain the precise meaning of fit in the context of SEM, even if the majority are aware that model fit, in general, describes how well a proposed model fits the empirical data. Model fit measures how closely the sample covariance matrix S, which is used to estimate the model, resembles the inferred covariance matrix, which is the form the data should have had if the hypothesized model were true in practice. The more similar or congruent the two covariance matrices are, the better the model fits overall. In our case, all the model fit indices indicate that the model has an excellent fit, i.e., the data fit the model well (Davvetas et al., 2020).

### 5.4 Testing of hypotheses

This study examined the impact of occupational stress on work–life balance. The structural model path coefficients show that the impact of occupational stress on work–life balance is negative and statistically significant (*ß* = −0.98, *t* = −2.969 *p* < 0.05), indicating a negative relationship and that for a one-unit increase in occupational stress, there is a 0.98-unit decrease in work–life balance, keeping other factors constant in the model. Overall, a negative relationship between occupational stress and psychological well-being was observed. Thus, H1: The impact of occupational stress on psychological well-being is statistically significant for information technology employees.

Similarly, the path coefficients for occupational stress and autonomy are not statistically significant (*ß* = −0.022, *t* = −0.378, *p* > 0.5); therefore, H1-1 is not supported. Furthermore, there was a negative association between occupational stress and positive relationships, as indicated by the path coefficients (Table 3). Therefore, H1-3 is not supported (Table 3). However, the relationship between occupational stress and environmental mastery is positive but statistically significant (*ß* = 0.058, *t* = 1.998, *p* < 0.05), supporting H1-2. Similarly, the impacts of occupational stress and self-acceptance were positive and not statistically significant (*ß* = 0.142, *t* = 5.259 *p* < 0.001), supporting H1-4.

**Table 3:** Testing of hypotheses (Path coefficients).

Relationship	Estimate	S.E.	C.R.	P	Result
Occupational stress → Psychological well-being	−0.98	0.33	−2.969	0.024	Supported
Occupational stress → Autonomy	−0.022	0.058	−0.378	0.706	Not supported
Occupational stress → Self-Acceptance	0.142	0.027	5.259	***	Supported
Occupational stress → Positive Relations	−0.019	0.037	−0.507	0.612	Not supported
Occupational stress → Environmental mastery	0.058	0.029	1.998	0.046	Supported
Work Interference with Personal Life →Autonomy	0.278	0.060	4.602	***	Supported
Work Interference with Personal Life →Self-Acceptance	0.234	0.029	8.136	***	Supported
Work Interference with Personal Life → Positive relations	0.274	0.040	6.909	***	Supported
Work Interference with Personal Life → Environmental Mastery	0.422	0.033	12.895	***	Supported
Work Personal Life Enhancement → Autonomy.	0.319	0.058	5.484	***	Supported
Work Personal Life Enhancement → Self-Acceptance	0.143	0.027	5.276	***	Supported
Work Personal Life Enhancement → Positive Relations	0.120	0.037	3.240	0.001	Supported
Work Personal Life Enhancement → Environmental Mastery	0.237	0.029	8.027	***	Supported
Personal Life Interference with Work → Autonomy	0.120	0.056	2.144	0.032	Supported
Personal Life Interference with Work → Self-Acceptance	0.176	0.027	6.595	***	Supported
Personal Life Interference with Work → Positive Relations	0.156	0.036	4.278	***	Supported
Personal Life Interference with Work → Environmental Mastery	0.035	0.028	1.247	0.212	Not supported

***Significant at <0.001 level.

The impact of work–life balance on psychological well-being is positive and statistically significant (*ß* = 0.947, *t* = 8.908, *p* < 0.001), supporting H2: the impact of work–life balance on psychological well-being is positive and statistically significant.

The structural model path coefficients indicate that the impact of work interference with personal life on the psychological well-being dimensions autonomy (*ß* = 0.278, *t* = 4.602, *p* < 0.001), self-acceptance (*ß* = 0.234, *t* = 8.136, *p* < 0.001), positive relations (*ß* = 0.274, *t* = 6.909, *p* < 0.01) and environmental mastery (*ß* = 0.422, *t* = 12.895, *p* < 0.001) is positive and statistically significant; therefore, H2-1: The impact of work interference with personal life on the psychological well-being dimensions autonomy, environmental mastery, positive relations and self-acceptance is positive and statistically significant.

Similarly, the impact of personal life interference with work on the psychological well-being dimensions of autonomy (*ß* = 0.120, *t* = 2.144, *p* < 0.05), self-acceptance (*ß* = 0.176, *t* = 6.595, *p* < 0.001), and positive relationships (*ß* = 0.156, *t* = 4.278, *p* < 0.01) was positive and statistically significant. However, the impact on environmental mastery (*ß* = 0.035, *t* = 1.247, *p* > 0.05) is positive and not statistically significant, which partially supports H2-2. The impacts of personal life interference with work on the dimensions of psychological well-being, autonomy, environmental mastery, positive relationships and self-acceptance are positive and statistically significant.

The impact of work personal life enhancement on the dimensions of psychological well-being and the structural model path coefficients show that autonomy (*ß* = 0.319, *t* = 5.484, *p* < 0.001), self-acceptance (*ß* = 0.143, *t* = 5.276, *p* < 0.001), positive relations (ß = 0.120, t = 3.220, p < 0.05) and environmental mastery (*ß* = 0.237, *t* = 8.0277, *p* < 0.001) are positive and statistically significant; therefore, supporting H2-3, the impact of work personal life enhancement on the dimensions of psychological well-being autonomy, environmental mastery, positive relations and self-acceptance is positive and statistically significant.

Tables 3, 4 show that some coefficients (ß coefficients or estimates) are not the same, some are high, and some are low. The variance that cannot be explained by every external predictor in the model is known as residual variance. If there is a correlation between the new exogenous variable and other endogenous variables, the coefficients vary between the covariance on and off models. Furthermore, the data were collected in a cross-sectional study, i.e., at a single time.

**Table 4:** Discriminant validity analysis (Fornell and Larcker, 1981) and HTMT analysis.

	CR	AVE	OSTR	WLB	PWB	OSRT	WLB	PWB
OSTR	0.917	0.582	**0.763**					
WLB	0.774	0.589	0.144*	**0.773**		0.22		
PWB	0.773	0.524	0.045	0.522***	**0.686**	0.078	0.068	

Values in bold are square root of Average Variance Extracted.

### 5.5 Validating higher-order constructs

**Figure 4:**
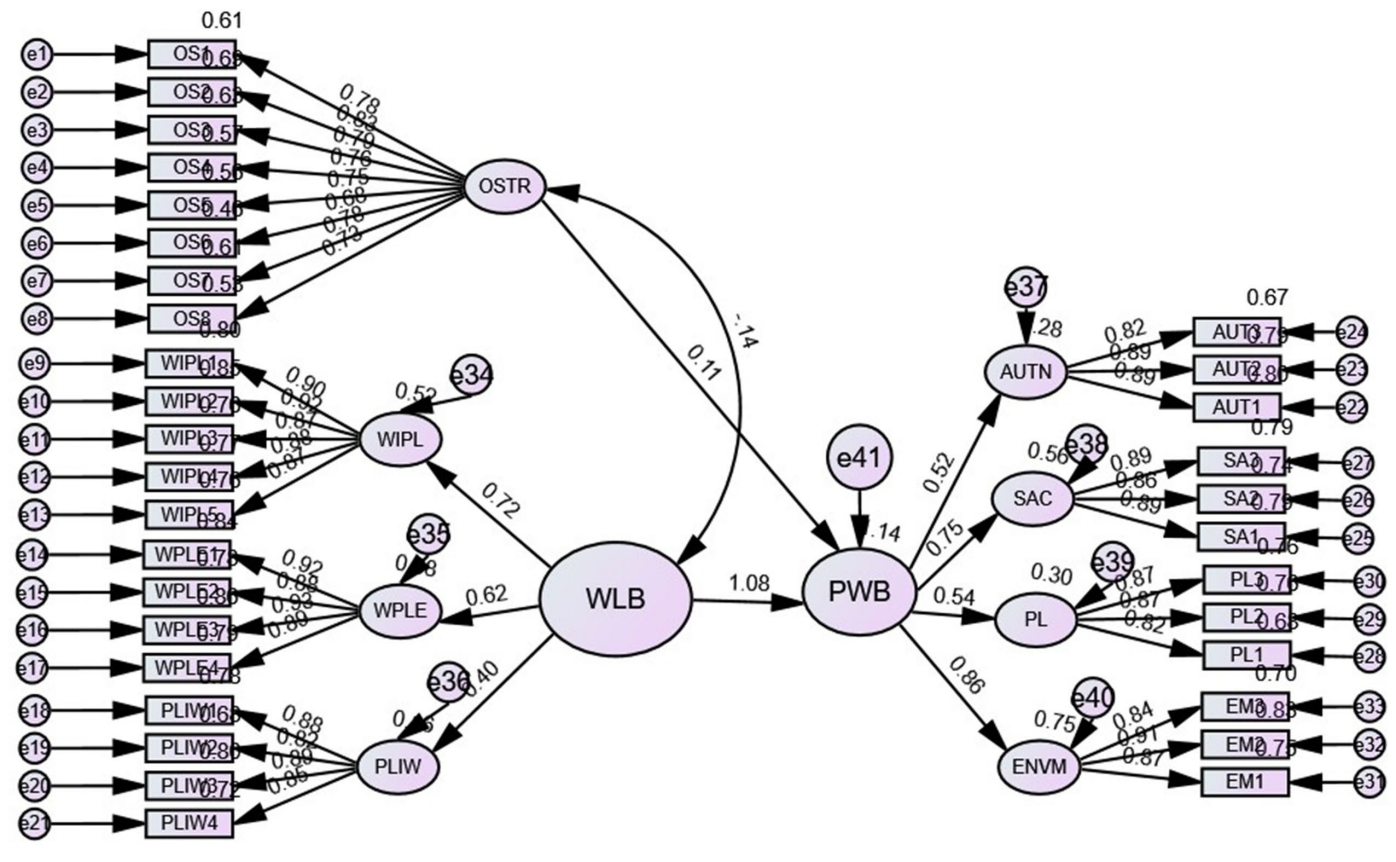
Structural model higher-order constructs.

In the second step, the higher-order constructs are validated. The three constructs of work–life balance and the four constructs of psychological well-being were modeled in higher order, with occupational stress modeled as a lower-order construct. First, the outer loadings of all the variables are calculated, and all the outer loadings are >0.7 (Figure 4) except one for occupational stress; however, this item is retained when the average loading for the construct is >0.7. As suggested by Ullah et al. (2021), the first step for measuring the reflective higher-order constructs is to measure multicollinearity issues in the reflective tolerance value of the independent variables; the tolerance values for the independent variables are >0.20; the variance inflation factor (VIF) values are less than the threshold limit of 4; and the eigenvalues for all the independent variables are not close to zero. Finally, the condition index values for all the independent variables are <15. Therefore, in the present study, no such multicollinearity issue was detected. Thus, further analysis was carried out.

The reliability, discriminant validity and convergent validity of the higher-order constructs were measured. The Cronbach’s alpha values for all the higher-order constructs are >0.70, indicating reliability and consistency. The composite reliability values for all the constructs are >0.7; the average variance extracted values are >0.5, and the constructs meet the (Fornell and Larcker, 1981) criterion (Table 4). All the HTMT ratios are less than 0.85 (Henseler et al., 2015).

### 5.6 Model fit statistics for the higher-order model

The hypothesized measurement model for higher-order constructs has three constructs: occupational stress (lower-order), psychological well-being (higher-order) and work–life balance (higher-order). The model fit is excellent, as evidenced by the model fit statistics. The model fit indices CMIN/df = 2.032, CFI = 0.962, TLI = 0.959, IFI = 0.963, NFI = 0.929, SRMR = 0.046, RMSEA = 0.045 and PClose = 0.966 indicate excellent structural model fit. The squared multiple correlations (R^2^) of 0.28 for autonomy, 0.56 for self-acceptance, 0.30 for positive relations and 0.75 for environmental mastery indicate that occupational stress and work–life balance explain 285% of the variance in autonomy, 56% of self-acceptance, 30% of positive relations and 75% of environmental mastery.

### 5.7 Common method bias

The inflation or depletion of the genuine correlation between the study’s observable variables is referred to as common method bias (CMB). Because respondents frequently answer questions with both independent and dependent variables, artificial inflation of covariance is possible. Using the common method latent factor and Harman’s single-factor test, this study evaluated common method bias.

#### 5.7.1 Harman’s single factor test

Confirmatory factor analysis was used to evaluate the model fit after the researchers loaded all the indicators onto a single factor. After verification, the model fit was not appropriate, ruling out common method bias.

#### 5.7.2 Latent common method factor

A latent construct with a direct relationship to each of the construct’s model indicators was employed by the researchers. A latent construct known as the common method was constructed. The model contained a direct correlation between each indicator in the model and the latent construct of the unobserved common method. A path is built from the common method construct to each indicator in the model, and then a constraint on all the relationships from the method factor is established to determine whether all of the elements have a common impact. The model was run with the latent common method variable, which has a direct relationship with all of the variables. The chi-square value for the CFA model was noted. The observed chi-square value is 892.617, with 467 degrees of freedom. With 628 degrees of freedom, the basic model’s chi-square with a latent factor is 888.700. The chi-square difference of 3.917 suggested the presence of common method bias. Since the CMB is so low and has little bearing on the study’s findings, it is not a significant problem in this empirical study.

### 5.8 Structural model assessment and hypothesis testing with higher-order constructs

After validating the measurement model of the higher-order constructs and lower-order constructs, the next step is to analyze the structural relationships among the higher-order constructs and to validate the hypotheses formed during the initial phase of the study. The multiple correlation (R^2^) value of 0.14% means that 14% of the variance in the dependent variable, psychological well-being, was explained by both the independent variables work–life balance and occupational stress.

The study determined the multidimensional nature of the impact of work–life balance on the multidimensional construct of psychological well-being. The impact of occupational stress is negative and statistically significant (ß = −0.76, *t* = 2.526, *p* < 0.05), indicating a negative relationship supporting H3, whereas the impact of work–life balance on psychological well-being is positive and statistically significant (*ß* = 0.947, *t* = 8.908, *p* < 0.001), indicating a positive relationship between work–life balance and psychological well-being. For a one-unit increase in work–life balance, there will be a 0.917 unit increase in psychological well-being (Table 5), supporting H4.

**Table 5:** Testing of hypotheses higher-order constructs.

Relationship	ß	SE	*t* value	*p* value	Result
H3: Occupational stress → psychological well-being	−0.076	0.030	−2.526	0.012	Supported
H4: Work-life balance → psychological well-being	0.947	0.106	8.908	<0.001	Supported

### 5.9 Mediation analysis

**Figure 5:**
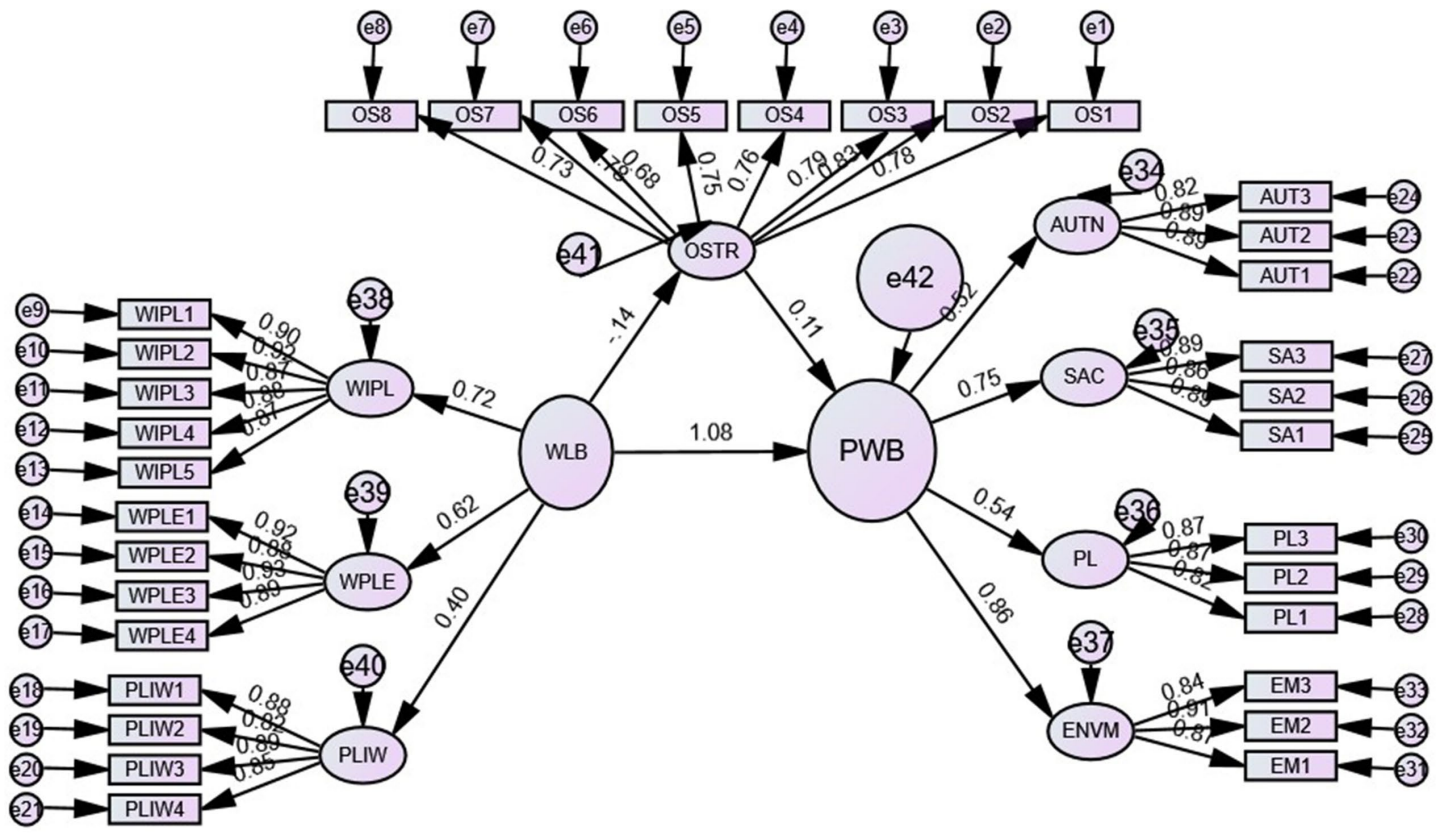
Mediation analysis.

The last proposition of the study is that occupational stress mediates the relationship between work–life balance and psychological well-being according to the path coefficients. The direct effect of work–life balance → psychological well-being is positive and statistically significant (*ß* = 0.629, *t* = 10.530, *p* < 0.001), and the indirect effect of work–life balance → occupational stress → psychological well-being is also positive (*ß* = 0.009, *p* < 0.010) and statistically significant. As the direct and indirect effects are positive and statistically significant (Table 6, Figure 5), H5: Occupational stress partially mediates the relationship between work–life balance and psychological well-being.

**Table 6:** Standardized total, direct and indirect effect- Bootstrap.

Relationship	ß	SE		Lower bound	Upper bound	*P* value	Result
Total effect							
Work-life balance → Psychological well –being	0.619	0.061	15.065	0.515	0.746	<0.001	
Direct effect							
Work-life Balance → Psychological well-being	−0.96	0.214	4.293	−0.032	−1.01	<0.001	
Indirect effect							
Work-life balance → Occupational stress → Psychological well-being	0.523	0.211		0.518	0.750	<0.001	Partial Mediation

## 6 Discussion

Occupational stress is a state of mind, and stress is perceived by an employee in his/her office setting, environment, policies, etc. The authors carried out this empirical study by surveying information technology employees who use metro rail as a mode of travel to commute regularly to and from home/office. The results indicate that occupational stress is statistically significant and impacts the psychological well-being of employees in general and their work–life balance in particular. Dalal (2022) carried out a similar study in public and private sector banks and reported statistically significant differences in the three constructs of occupational stress, work–life balance and psychological well-being in the context of public–private sector banking employees. Our results are consistent with the findings of this author. Work–life balance significantly impacts employee productivity, with imbalances affecting job autonomy, performance, and organizational citizenship behavior. A study in the Philippines revealed that implementing a compressed workweek (CWW) reduces stress, enhances work–life balance, and improves productivity, potentially benefiting employers considering the institutionalization of CWW (Bhende et al., 2020; Brough et al., 2020; Haar and Brougham, 2022; Paje et al., 2020). Our results support the findings of these authors’ outcomes.

Karani et al. (2022) examined the relationship between work–life balance and psychological distress by surveying 400 employees working in the banking sector. The PLS-SEM results indicate that family satisfaction, role conflict, and work overload are the main factors influencing occupational stress and psychological well-being. In another study, the authors investigated the influence of psychological well-being and work–life balance issues in the context of women’s mobility. The results reveal a negative relationship between psychological welfare and not having a work–life balance and between psychological welfare and interorganizational mobility. The authors also reported that psychological well-being is an important factor in managing work–life balance (Kadir et al., 2021). A study by Shange (2022) revealed that 89% of academics at a University of Technology in KwaZulu-Natal, South Africa, work long hours and have less time for family, negatively impacting productivity and psychological well-being. Additionally, 20% of academics still acquire their minimum qualifications, indicating a need for improved work–life balance. Potts et al. (2023) carried out a meta-syntheses to systematically identify and draw together qualitative research evidence on coaches’ experiences of stressors, primary appraisals, emotions, coping, and PWB. The results demonstrate the wide range of stressors that coaches may encounter, the effects that coaches’ evaluations have on PWB, and the coping mechanisms that coaches might employ to promote adaptability. By doing so, the meta-synthesis broadens our comprehension of coaches’ experiences with PWB and stress transactions. Our results support the authors’ findings.

Aderibigbe (2021) and Tommasi et al. (2022) reported on the stress, psychological well-being and psychological disease, and occupational stress of Italian firexhters and Nigerian graduate employees, respectively. Studies have reported that occupational stress and work–life balance negatively impact the psychological well-being of employees and have suggested mitigating occupational stress and enhancing work–life balance. Gulsher and Siddiqui (2021) investigated the effects of an organizational ethical climate on work–life balance and psychological well-being by surveying the employees of manufacturing companies in Pakistan. The PLS-SEM results indicated that quality of service and employee caring positively and significantly impacted the well-being and competence of the employees. Work–life balance is the major contributor to the psychological well-being of employees. Our results are consistent with and similar to those of the cited studies. In our empirical study, we used AMOS for SEM analysis, which also resulted in similar findings.

Javaid et al. (2023) investigate the novel approach of using work organization and job content (WOJC) as a higher-order construct, which is one of the domains of the Copenhagen Psychosocial Questionnaire (COPSOQ), examining its relationship with sleeping difficulties and somatic stress, as well as the moderating effect of leadership quality. The structural equation modeling technique was used to produce the desired range of outcomes. The findings show that the WOJC has a considerable negative influence on both psychological (sleeping issues) and physiological (somatic stress) health parameters among poultry workers. Leadership quality did not influence the connection between the WOJC score and physiological health indicators, but it did affect the relationship between the WOJC score and psychosocial health factors. The study looked at the burnout–depression distinction using the Occupational Depression Inventory (ODI), a recently created assessment of work-related depressive symptoms. The ODI was used to assess work-related depression symptoms. The Shirom-Melamed Burnout Measure (SMBM) and Oldenburg Burnout Inventory (OLBI) were used to evaluate burnout symptoms. The SMBM defines burnout as a syndrome that includes physical fatigue, cognitive weariness, and emotional depletion. The OLBI defines burnout as a combination of tiredness and disengagement. Confirmatory factor analysis revealed that the factors underlying burnout components correlated more strongly with the occupational depression factor than with each other, casting doubt on the syndromal unity of burnout. Furthermore, the factors underlying burnout and occupational stress are related to a similar higher-order factor (Sowden et al., 2022). Ho and Chan (2022) described psychological capital (PsyCap) as a higher-order core construct that includes hope, efficacy, resilience, and optimism. The purpose of this study is to look at the effects of PsyCap on the well-being of social workers and to investigate the underlying processes of this link. The quantitative results revealed that PsyCap was positively linked with job satisfaction and positive affect and was adversely associated with negative affect and psychological and physical suffering.

## 7 Conclusion

The survey results reveal that employees are happy to travel in metros even during the COVID-19 pandemic, following the COVID-19 protocols of the Government of India. The employee further said that their travel time has considerably decreased because road travel is costly, with traffic jams and time consumption. The employees expressed that they could reach their offices/Homes in a timely manner because metro travel is timely and secure and causes minimum stress. The metro rail will have centralized AC, which is more pleasant than public transport. The employees felt that at least 2 h of free time are at their disposal and can be utilized for other purposes, such as attending family work, finishing pending work, and visiting relatives. One employee commented, “The metro travel changed my life, I speak to my relatives or friends during the journey, or spend my journey time in a relaxed mood reading a book of their choice, watching movies to get relief which otherwise she could not have done so through public transport.” This reveals that commuters lead a more comfortable and qualitative life, leading to minimal stress through economic and state-of-the-art public transportation, which facilitates the fastest yet safest journey. Metro travel is pollution free, and all weather transport is safe and enjoyable.

### 7.1 Theoretical contributions

Researchers can model a construct on a more abstract dimension (called a higher-order component) and its more concrete subdimensions (called lower-order components) via higher-order constructs, also called hierarchical component models, in structural equation modeling (SEM). This study addresses a prevalent misunderstanding in researchers’ reliability and validity assessments by offering a technique for assessing higher-order structures in SEM via repeated indicators and two-stage techniques. The paper offers recommendations for academics, marketing researchers, and practitioners by demonstrating the specification, estimation, and validation of reflective-reflective higher-order structures via SEM. Organizational practices to reduce stress and improve employee work–life balance and psychological well-being in general and the IT sector in particular will be more practically applicable as a result of testing the constructs with both higher- and lower-order constructs and evaluating the results.

### 7.2 Practical implications

The authors investigated the direct relationship between work–life balance (WLB) factors and psychological well-being using occupational stress as a model. The overload supports all of the hypotheses. This study contributes to the literature by examining the effects of work–life balance on psychological well-being in the context of occupational stress. This study also provides empirical evidence to HR managers for developing effective HR policies to reduce occupational stress and improve work–life balance, which leads to improved psychological well-being. As a result, employees can continue to improve their personal and professional lives. Another important aspect of well-being is social wellness, which is primarily determined by job satisfaction and workplace culture. In this context, employee recognition can have a significant effect. Encouragement from leaders, supervisors, and coworkers to recognize employees’ achievements can have a significant effect on motivation, making people feel valued and, ultimately, satisfied with their jobs. In the IT sector, employees’ psychological well-being and mental health are significantly influenced by work–life balance, and the IT industry should prioritize and develop policies to increase work–life balance to mitigate the negative effects of psychological well-being. The findings emphasize how crucial it is to take work–life balance into account and provide chances for better psychological well-being work–family balance when developing teleworking plans. By offering data that enable employers to optimize health advantages and minimize the hazards of working from home during (and possibly after) the pandemic, this study advances knowledge and practice. Organizations should develop and inculcate flexible work schedules for employees who can use metro rails to reach their destination when the rush speed does not peak, as metro travel considerably reduces the commute time of employees.

The authors further suggest Flextime versus work intensity, Flexplace versus space constraints, Technologically possible work arrangements versus isolation and stress, and Family-friendly work arrangements versus the intensity of housework and care. We draw attention to the critical role that HRD professionals may play in helping workers align their expectations and experiences with remote employment.

### 7.3 Limitations and future directions

The study was conducted between April and mid-June 2021, when the second wave of the COVID-19 pandemic peaked. We distributed the research instrument, a survey form with a hard copy link slip, to approximately 20,000 people who were moving from a metro station to a station. Some travelers refused to collect the link slip, which contained the link to the questionnaire. Some people were given mobile numbers to share the link. The study is geographically limited to the Hyderabad Metro city. The authors strongly recommend conducting similar studies across Indian metro stations. Although the study was conducted in Hyderabad, the results can be generalized because the authors took great care in measuring internal consistency and reliability, as well as fulfilling all of the assumptions required for general linear model univariate analysis. Women commuters are more enthusiastic about submitting responses. Some commuters expressed concern about the lack of security near the staircase and lift, as intruders could gain access to the entire area near the toilets that is not manned by security. To advance our understanding of these topics, qualitative and/or longitudinal research is necessary. To reduce stress, promote PWB, and facilitate positive evaluations and emotions, such studies should be used to develop interventions that are suitable for various coaching populations (e.g., working parents and part-time coaches). Survey research is prone to sampling bias. However, this bias was mitigated by gathering data from different information technology companies while distributing the questionnaire link. We ensure that our procedure gives every member of the target population an equal chance to be included in the sample group. Additionally, our survey always included a statement assuring respondents that their responses would remain anonymous and be utilized exclusively for the study’s objectives. “This survey is anonymous. Nobody will be able to determine whether or not you took part in the study, nor will they be able to identify you or your responses.” Our sample is large, and the results can be generalized to some extent, i.e., IT sector employees.

The authors suggest that longitudinal studies collect data from the IT industry from different states of India to include diverse geographic locations and among different groups, including multicultural respondents. The authors also recommend carrying out studies in different sectors, such as the banking and healthcare sectors, to further advance and generalize the studies.

## Data Availability

The datasets presented in this study can be found in online repositories at https://figshare.com/s/42935f49530037fcd275.
